# Small changes in rhizosphere microbiome composition predict disease outcomes earlier than pathogen density variations

**DOI:** 10.1038/s41396-022-01290-z

**Published:** 2022-07-22

**Authors:** Yian Gu, Samiran Banerjee, Francisco Dini-Andreote, Yangchun Xu, Qirong Shen, Alexandre Jousset, Zhong Wei

**Affiliations:** 1 Joint International Research Laboratory of Soil Health, Jiangsu Provincial Key Lab for Organic Solid Waste Utilization, Jiangsu Collaborative Innovation Center for Solid Organic Waste Resource Utilization, National Engineering Research Center for Organic-based Fertilizers, Nanjing Agricultural University, Nanjing, PR China; 2 College of Food Science and Light Industry, Nanjing Tech University, Nanjing, PR China; 3 Department of Microbiological Sciences, North Dakota State University, Fargo, ND USA; 4 Department of Plant Science, The Pennsylvania State University, University Park, PA USA; 5 Huck Institutes of the Life Sciences, The Pennsylvania State University, University Park, PA USA

**Keywords:** Microbial ecology, Microbial ecology

## Abstract

Even in homogeneous conditions, plants facing a soilborne pathogen tend to show a binary outcome with individuals either remaining fully healthy or developing severe to lethal disease symptoms. As the rhizosphere microbiome is a major determinant of plant health, we postulated that such a binary outcome may result from an early divergence in the rhizosphere microbiome assembly that may further cascade into varying disease suppression abilities. We tested this hypothesis by setting up a longitudinal study of tomato plants growing in a natural but homogenized soil infested with the soilborne bacterial pathogen *Ralstonia solanacearum*. Starting from an originally identical species pool, individual rhizosphere microbiome compositions rapidly diverged into multiple configurations during the plant vegetative growth. This variation in community composition was strongly associated with later disease development during the later fruiting state. Most interestingly, these patterns also significantly predicted disease outcomes 2 weeks before any difference in pathogen density became apparent between the healthy and diseased groups. In this system, a total of 135 bacterial OTUs were associated with persistent healthy plants. Five of these enriched OTUs (*Lysinibacillus*, *Pseudarthrobacter*, *Bordetella*, *Bacillus*, and *Chryseobacterium*) were isolated and shown to reduce disease severity by 30.4–100% when co-introduced with the pathogen. Overall, our results demonstrated that an initially homogenized soil can rapidly diverge into rhizosphere microbiomes varying in their ability to promote plant protection. This suggests that early life interventions may have significant effects on later microbiome states, and highlights an exciting opportunity for microbiome diagnostics and plant disease prevention.

## Introduction

**Fig. 1:**
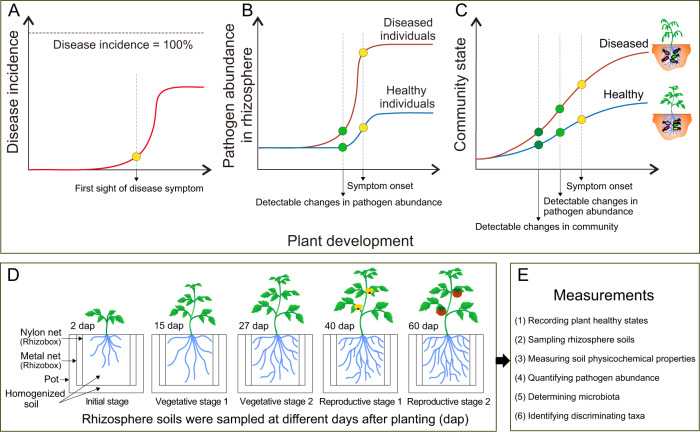
Hypotheses and experimental design. **A** Scenarios of disease incidence in the pathogen-only experiment. Plants are expected to die after pathogen inoculation, while (generally) not all plants show disease symptoms, and some plants survived up to the end of the experiment (binary disease outcome). **B** Scenarios of pathogen abundance in the rhizosphere of the pathogen-only experiment. Although plant individuals are exposed to the same initial abundance of the pathogen in the rhizosphere, compared to healthy plants, the diseased plants often have higher pathogen loads in the rhizosphere. Changes in pathogen abundance between healthy and diseased plants are detectable prior to the onset of disease symptoms. **C** Schematic figure of the hypotheses. Soil community state gradually changes as plants grow. We hypothesize that the microbiome can diverge toward different configurations out of an initial constant species pool, later affecting plant protection against disease. **D** Schematic figure of the experimental design. We used the rhizobox system [6] for nondestructive repeated sampling of individual plants at different growth stages. Soils associated with 54 plant individuals at the initial stage (IS; 2 days after planting (dap)), vegetative stage 1 (VS1; 15 dap), vegetative stage 2 (VS2; 27 dap), reproductive stage 1 (RS1; flowering stage; 40 dap), and reproductive stage 2 (RS2; fruiting stage; 60 dap) were collected. **E** We recorded the disease incidence, sampled rhizosphere soils, measured soil physicochemical properties, quantified pathogen abundance, determined bacterial community composition, and identified the discriminating OTUs associated with healthy and diseased plants.

Most agricultural soils are infested with some level of plant pathogens. Soilborne pathogens can persist for several years before infecting a new host and marginally respond to pesticides, resulting in major yield losses worldwide [1, 2]. Understanding the disease dynamics is essential to prevent pathogen spread and yet such often remains elusive. One particularly salient feature of soilborne diseases is the binary outcome of infections. In many outbreaks, plants either remain healthy or get severely ill and die. Part of this distribution has been typically attributed to heterogeneity in local conditions including the pathogen abundance in soil [3], the genetic background of the pathogen [4], the host plant genotype [5], the soil and plant-associated microbiome, and/or soil physicochemical properties [6, 7]. However, the hypothesis of heterogeneity is challenged by observations that a binary disease outcome also occurs in homogeneous conditions (Fig. 1A), with pathogen density remaining low in some individuals, while rapidly rising and causing disease in others (Fig. 1B). The rhizosphere microbiome is crucial for plant health [8], and it is known to change in composition and function during plant development [9]. In a previous study, we demonstrated that a small variation in the local species pool had cascading effects on the rhizosphere microbiome assembly. This resulted in plant health or death in the presence of a pathogen [6]. In this work, we tested whether these alternative states of the rhizosphere microbiome (i) form out of an initially homogenized soil; and (ii) affect the level of plant protection against a soilborne pathogen (Fig. 1C). These hypotheses are linked to the widespread presence of bifurcations in ecosystem development, where different alternative states emerge out of a homogenous configuration [10].

Pathogen infection is often reported as density-dependent. While infection requires a threshold density, soil pathogen density is a poor predictor for disease onset in field conditions [6]. Soil homogenization removes this confounding factor by setting pathogen variation to zero. New methods are thus needed for early disease diagnosis allowing for the detection of vulnerabilities before the disease outbreak. In this sense, microbiome diagnostics can offer a possible solution. For instance, in humans, numerous studies have revealed the close ties between microbiome composition and various diseases (e.g., colorectal cancer, cirrhosis, arthritis, and irritable bowel syndrome) [11]. As a result, there is a growing interest in microbiome research toward advancing disease diagnostic and therapies [12]. For example, microbial biomarkers contributing to human health have been discovered and further applied as probiotic agents to improve clinical interventions [13, 14]. Such information is particularly scant in plant-microbiome studies. Furthermore, most studies in the field of biomarkers are correlative as they compare microbiome compositions when the disease has already been set on. The observed correlations may therefore be confounded by the presence of species associated with the diseased environment but without causal effect on disease [15] or even generate erroneous predictions, as the species that survived in the diseased environment are often antagonistic to the pathogen [16]. Thus, the development of longitudinal studies may solve the causal relationship between microbiome assembly and disease onset. As such, we used this approach in the past to demonstrate the importance of local variation of the founding species pool for microbiome dynamics [6] (Fig. S1). In the present study, we modified the setup to remove the initial variance in microbial and physicochemical properties. This allowed us to study whether heterogeneity in plant-microbiome composition and function can emerge out of an initially homogeneous species pool. This variation can be further explored in line with plant phenotypic responses (i.e., health and disease), allowing for inference of causal interactions between microbiome assembly and plant health.

The present study scrutinizes microbiome development together with disease susceptibility to the pathogenic bacterium *Ralstonia solanacearum*, the causative agent of bacterial wilt disease affecting more than 200 crops [2]. Soilborne pathogens need to invade the root microbiome and spread as a saprophyte until reaching a trigger population density, prior to causing infection. Thus, pathogen success is a direct function of its interaction with the rhizosphere microbiome [17]. In order to elucidate the causal relationships between microbiome assembly and disease, we used a longitudinal study with tomato plants growing in a natural homogenized soil infested with *R. solanacearum*. By using this nondestructive sampling system (Fig. S1), we were able to regularly assess microbiome assembly for each plant, long before the appearance of wilt disease symptoms (Fig. 1D). We recorded the plant healthy status, sampled rhizosphere soils, measured soil physicochemical properties, quantified soil pathogen abundance, profiled bacterial community composition, and identified discriminating taxa associated with disease outcomes (Fig. 1E). We expected that the rhizosphere microbiome assembly will not follow a linear process. Instead, due to the high level of facilitation between rhizosphere bacteria [16, 18], we expected that microbiome assembly may take a turn at any moment and assemble into a range of alternate configurations out of an initially homogenous soil community [19, 20]. We further expected these variations in microbiome states to alter competitive interactions with an invading pathogen, thereby resulting in differences in disease development at later stages of plant development (Fig. 1C).

## Materials and methods

### Meta-analysis of bacterial wilt incidence in tomato

We used *Web of Science* to search for published peer-reviewed articles using the keywords “bacterial wilt of tomato” to investigate the prevalence of binary infection outcomes. Data were extracted according to the following criteria: (i) studies from 2010 to 2020 with tomato as the host plant were selected; (ii) disease incidences of tomato plants grown in water or sterile soil were not included in the dataset; (iii) since we only focused on well-controlled experiments, and complex variable environmental factors exist under field conditions, the disease incidences of tomato grown under field experiments were also excluded from the dataset; (iv) for studies that manipulated multiple factors (e.g., biocontrol agent, specific genes of the pathogen, and management practice), only data from controls and regular fertilization treatments were included; (v) when articles reported multiple independent manipulative experiments (e.g., experiments at separate sites), each experiment was considered as an independent study and included in the dataset [21]; (vi) when articles reported bacterial wilt incidence at different time-points, only the latest time-point was considered; (vii) when data were only reported as figures, the data means were extracted using GetData-Graph Digitizer (www.getdata-graph-digitizer.com). In total, this meta-analysis included 132 observations from 58 articles (External Databases S1).

### Microcosm experiment

The soil was collected at a depth of 0–20 cm from 15 random sites in a tomato field in Qilin, Nanjing, China. This site is historically infested with *R. solanacearum*. The collected soil was processed to remove plant debris and sieved (2 mm for middle-layer nylon bags of the rhizobox and 4 mm for use in the greenhouse experiment; see below) without air-drying to preserve the living soil microbiome.

We used a previously developed mesocosm system (“rhizobox”) [6] to repeatedly collect rhizosphere soil from individual plants without damaging the plant root system (Fig. S1). This system allows for tracking the successional changes in the rhizosphere microbiome composition throughout the plant developmental stages, and for quantifying the dynamics of pathogen abundance and soil physicochemical properties. In contrast to the original setup that employed sterilization by gamma radiation of the soil in the nylon bags [6], we thoroughly homogenized all the soils in bag and pot to ensure that each plant was exposed to an initially homogeneous soil microbiome, soil physicochemical properties, and pathogen abundance. We provide a detailed description of the mesocosm system in the Supplementary Information (Appendix S1), and elsewhere [6].

### Experiment design and sampling strategy

Tomato seeds (*Solanum lycopersicum* cv. Hezuo 903) were surface-sterilized in 3% NaClO (v/v) for 5 min, rinsed four times with sterile distilled water, germinated in the dark for 2 days, and sown in a nursery substrate (Huaian Agricultural Technology Development Ltd., Huaian, China). After incubation in the greenhouse at 28 ± 3 °C for 3 weeks, tomato seedlings with similar sizes were gently washed. One seedling was planted in the root compartment of each rhizobox, previously filled with 1.2 kg of homogenized soil (<4 mm). The rhizoboxes were buried in pots containing 4 kg of the same batch of homogenized soil (<4 mm). A total of 54 pots were randomly placed and maintained in the greenhouse under ambient conditions suitable for tomato growth (28 ± 3 °C).

To collect rhizosphere soil in a nondestructive manner, three of the middle-layer nylon bags were randomly collected at each sampling time and pot samples were pooled into one composite sample, placed on ice, and transported to the laboratory (<6 h). The collected soils were stored at −80 °C until further processing. Samples were collected from 54 individual plants at the initial stage (IS; 2 days after planting (dap)), vegetative stage 1 (VS1; 15 dap), vegetative stage 2 (27 dap), reproductive stage 1 (RS1; flowering stage; 40 dap), and reproductive stage 2 (RS2; fruit developmental stage; 60 dap) (Fig. 1D). The growth state of the tomato roots in the root compartment was checked routinely to ensure that the root compartment was densely rooted at 15 dap and onwards. This is important to ensure that the collected samples correspond to the rhizosphere. In this system, samples collected 2 dap were treated as bulk soil.

The disease index was recorded separately for each plant on a scale of 0–4 (0: no wilting; 1: 1–25% of leaves wilted; 2: 26–50% of leaves wilted; 3: 51–75% of leaves wilted; 4: 76–100% of leaves wilted). Disease incidence was then calculated as = [∑ (number of diseased plants in given disease index × given disease index) × (total number of plants × highest disease index)^−1^] × 100% [22]. Fifty percent of the tomato plants (i.e., 27 individual plants) showed wilting symptoms at 60 days after planting (Fig. S2A). The plants were incubated for an additional week (67 days in total) to ensure that none of the symptomless plants developed any wilt symptoms. After 67 days, all tomato plants were collected and tested for the density of *R. solanacearum* in their stem crowns. This was carried out by macerating 5 g of stem crown tissues in 45 mL sterile 0.9% NaCl followed by serial dilution and plating on *R. solanacearum* semi-selective medium (SMSA) [23]. *Ralstonia solanacearum* is often detected in symptomless plants harboring significant pathogen populations [4]. This state is known as latent infection. As a result, plant samples were separated into three disease outcomes: (i) healthy plants showing no wilt symptoms and tested negative for *R. solanacearum*, (ii) diseased plants showing wilt symptoms and tested positive for *R. solanacearum*, and (iii) latently infected plants showing no wilt symptoms and tested positive for *R. solanacearum*. At the end of the greenhouse experiment, we found 13 healthy plants, 14 latently infected plants, and 27 diseased plants. As latent infection may both represent an upcoming disease or an asymptomatic infection, we opted for excluding them from further analyses and only assessed healthy and diseased plants. For that, ten healthy and ten diseased plants were randomly selected and the associated soil samples (2 disease outcomes and 5 developmental stages resulting in 100 samples) were used for further analyses (Fig. S2B).

### Soil DNA extraction and pathogen quantification

Total genomic DNA was extracted from 400 mg of soil using the PowerSoil DNA Isolation Kit (QIAGEN, Hilden, Germany). DNA concentrations were determined using a NanoDrop spectrophotometer (ND2000, ThermoScientific, DE, USA). Aliquots of the extracted DNA samples were used for quantitative PCR analysis to determine the absolute abundance of *R. solanacearum*, and for PCR amplification prior to the bacterial small-subunit (SSU) rRNA gene high-throughput sequencing.

Quantitative PCR analysis was performed using the specific *R. solanacearum* primer set Rsol_*fli*C [24]. Each sample was measured in triplicate on a 7500 Fast Real-Time PCR System using the SYBR Premix Ex Taq Kit (Takara, Dalian, China) (Applied Biosystems, CA, USA), according to the manufacturer’s instructions (Table S1). We did not include reference DNA as an internal standard to the isolated DNA and this can introduce possible uncontrolled biases in pathogen quantification. In this study, we generated a standard curve using a plasmid (pMD 19-T vector, Takara, Dalian, China) containing the *fli*C gene of the *R. solanacearum* strain QL-Rs1115 [25].

### Bacterial small-subunit rRNA gene amplification and amplicon sequence analysis

The partial SSU rRNA gene was amplified using the primer set 563F and 802R [26] with attached Illumina flow cell adapters and sample-specific 8-bp barcodes, according to previously described PCR conditions (Table S1) [27]. Each sample was amplified in triplicate to minimize the bias of PCR amplification and pooled before purification using the AxyPrep PCR Clean-up Kit (Axygen Biosciences, CA, USA). The purified PCR products were quantified with QuantiFluor-ST (Promega, WI, USA), and sequencing libraries were constructed as previously described [28]. Sequencing was carried out on an Illumina MiSeq platform (2 × 250 bp) (Biozeron Biological Technology Co. Ltd., Shanghai, China).

Quality control of sequence reads was performed using the UPARSE pipeline [29]. Briefly, paired-end reads were assembled and trimmed (maximal expected errors of 0.25, reads length >200 bp). Singletons were removed prior to clustering into OTUs at 97% of nucleotide similarity, followed by filtration of chimeras using UCHIME [30]. Sampling effort was equalized to the depth of the smallest sample (23, 639 reads). We also validated the OTU-based results using a 100% sequence similar zero-radius OTUs (zOTUs) [31]. This was carried out by examining the correlation between pairwise dissimilarities (Bray–Curtis index) obtained for the OTU-based and zOTU-based methods. Filtered sequences were clustered into zOTU using the UNOISE 3 algorithm implemented in USEARCH. Taxonomic assignments of the OTUs were obtained using the Ribosomal Database Project (RDP) pipeline (Taxonomy 18) [32] with a confidence threshold of 80%. Raw sequences were deposited in the DDBJ SRA under the accession numbers PRJNA299538 and PRJNA806399.

### Characterization of soil physicochemical properties

Soil physicochemical properties were determined using standard operating procedures. Electrical conductivity and pH were measured using a conductivity meter (DDS-307A, Rex, Shanghai, China) and a Sartorius PB-10 pH meter (Göttingen, Germany), respectively. NH_4_^+^-N and NO_3_^−^-N were measured by KCl extraction using a Lachat Flow Injection Analyzer (AutoAnalyzer3-AA3, Seal Analytical, WI, USA). Available phosphorus (P) was determined using the molybdenum blue method [33]. Total carbon (C) and nitrogen (N) were quantified using an elemental analyser (Vario EL III, Elementar, Hanau, Germany). Dissolved organic carbon and organic nitrogen were determined using a Liqui TOC elementar analyzer (Elementar Co., Hanau, Germany). Each soil physicochemical property was transformed to range between 0 to 1 and all the measured properties were used for principal component analysis (PCA) to visualize variations across samples.

### Rhizobacteria isolation, taxonomic assignments, and isolate-OTU match

We conducted a separate greenhouse experiment to isolate rhizobacteria from tomato plants at vegetative stage 2 (i.e., 27 dap). Tomato seeds (cv. Hezuo 903) were surface-sterilized, germinated, and sown in nursery substrate as described above. Three weeks after sowing, seedlings were planted in pots (not rhizobox) containing 2 kg of homogenized soil (<4 mm). The soil was the same as used above and each pot contained one seedling. Twelve tomato plants were destructively sampled at 27 dap and none of these individuals showed disease symptoms. To harvest the rhizosphere soil, the excess soil was first removed from the tomato roots by shaking and the remaining soil adhered to the roots (rhizosphere soil) was collected. Prior to bacteria isolation, soils of three individual plants were randomly pooled (resulting in four soil replicates). This strategy was used to reduce the number of soil samples and increase the depth of rhizobacteria isolation in each sample. The isolation started with soil suspensions serially diluted and plated on 1/10 tryptone soy agar (1.5 g L^−1^ tryptone, 0.5 g L^−1^ soytone, 0.5 g L^−1^ NaCl, and 15 g L^−1^ agar). After 2 days of incubation at 30 °C, a total of 137 isolates (32-37 isolates per replicate, *n* = 4) were randomly selected and purified.

Bacterial isolates were taxonomically classified based on the SSU rRNA gene sequence using a standard procedure [16]. Briefly, the total genomic DNA of each isolate was extracted using EZNA bacterial DNA isolation kit (Omega Bio-tek, Norcross, GA, USA), following the manufacturer’s instructions. Amplification targeting the bacterial SSU rRNA gene was carried out using the primer set F27 and R1492 [34] (Table S1). The PCR products were sequenced at Songon Biotechnology Co. Ltd (No. 698, Xingmin Road, Songjiang Zone, Shanghai, China). The SSU rRNA sequences were classified using the NCBI and RDP pipeline. The obtained 137 isolated bacteria were taxonomically affiliated to the phyla *Pseudomonadota* (formerly *Proteobacteria*), *Actinomycetota* (formerly *Actinobacteria*), *Bacillota* (formerly *Firmicutes*), and *Bacteroidota* (formerly *Bacteroidetes*) (Table S2).

We mapped the full-length SSU rRNA gene sequences of the bacterial isolates to the OTU representative sequences obtained via high-throughput sequencing. For that, bacterial SSU rRNA gene sequences of the isolates were trimmed to the same region of the high-throughput sequencing data using the RDP pipeline. Then, sequences were mapped to the OTU representative sequences at ≥97% sequence similarity using UPARSE. Out of the 137 bacterial isolates, 118 isolates (86%) matched 31 OTUs retrieved by high-throughput sequencing, and only 19 isolates (14%) were not matched with a representative sequence of OTUs. Out of the 31 matched OTUs, 5 were enriched by healthy plants at VS2, while the remaining showed non-significant variations in relative abundance between healthy and diseased plants.

### Effects of isolated bacteria on bacterial wilt disease

One additional greenhouse experiment was conducted to test the disease suppression ability of each retrieved isolate with corresponding OTU matches. This included the five healthy-plant-enriched isolates/OTUs (Fig. 4E), and ten randomly selected non-discriminating isolates/OTUs (Table S3). Tomato seeds (cv. Hezuo 903) were surface-sterilized and germinated as described above. Each germinated seed was sown in pots containing 80 g of nursery substrate. The pots were incubated under ambient conditions suitable for tomato growth (28 ± 3 °C). After sowing for 25 days, each isolated bacteria were applied individually per treatment at a cell density of 1 × 10^6^ CFU g^−1^ of nursery substrate. One week after inoculation of the isolated bacteria, *R. solanacearum* QL-Rs1115 was inoculated at a cell density of 2 × 10^6^ CFU g^−1^ of nursery substrate. For the control treatment, only the pathogen was inoculated. For each treatment, 18 individual plants were used (*n* = 18), resulting in a total of 288 plants. The disease index was recorded 25 days after pathogen inoculation.

### Statistical analyses

All statistical analyses were carried out in R-4.0.3 [35]. Nonparametric Mann–Whitney test and Student’s *t* test were used to compare mean differences associated with disease outcomes. PCA was conducted using the “prcomp” command available in the vegan package [36]. The composition of bacterial communities was ordinated by principal coordinates analysis (PCoA) based on Bray–Curtis distances. Differences in community composition and soil physicochemical properties between disease outcomes were compared using permutational multivariate analysis of variance (PERMANOVA) using the vegan package. Linear discriminant analysis (LDA) was used to explore the most discriminating OTUs between health conditions using LEfSe [37]. A factorial analysis of variance (ANOVA, Tukey’s HSD test) was used to compare the mean differences between disease indexes.

## Results

### Consistent binary outcome of bacterial wilt disease

**Fig. 2:**
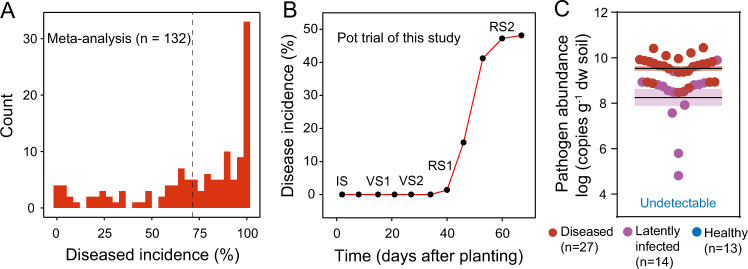
Contrasting disease outcomes of tomato individuals under pathogen pressure. **A** Frequency distribution of bacterial wilt incidence of tomato, based on a meta-analysis. Tomato plant individuals were exposed to treatment with the pathogen-only, and certain individuals survived up to the end of the experiment. The dashed line indicates an average of 71.4%. **B** Progression of the bacterial wilt disease incidence of the 54 tomato plant individuals in this study. Soil sampling was ended 60 days after planting and the plants were incubated for one additional week to ensure that none of the symptomless plants developed wilt symptoms (67 days in total). IS initial stage, VS1 vegetative stage 1, VS2 vegetative stage 2, RS1 reproductive stage 1, RS2 reproductive stage 2. **C** *R. solanacearum* abundance in the stem crown tissue of healthy, latently infected, and diseased tomato plants at the end of the greenhouse experiment (67 dap). The shaded area shows mean ± standard error. Pathogen abundance in the stem crown tissue of latently infected and diseased tomato plants was compared based on a nonparametric Mann–Whitney test.

Based on the dataset consisting of 132 observations published between 2010 and 2020, we found the incidence of bacterial wilt in tomato to range from 0 to 100%, with an average of 71.4% in greenhouse experiments (Fig. 2A). Importantly, in 105 of the 132 greenhouse observations, part of the plants remained healthy even under high disease pressure. These findings point to the existence of a binary disease outcome even under well-controlled plant growth conditions. We found that the earliest bacterial wilt disease symptoms tend to appear at RS1, with the disease incidence increasing sharply in the following weeks (Fig. 2B, Fig. S2A). Based on the wilt symptom and the pathogen density in the stem crown (Fig. 2C, Fig. S2A), plants were classified as diseased (*n* = 27), latently infected (*n* = 14), and healthy (*n* = 13) at the end of the greenhouse experiment. A significantly higher number (4.8-fold higher, *p* < 0.001, nonparametric Mann–Whitney test) of *R. solanacearum* were detected in the crown tissue of diseased plants when compared to latently infected ones (Fig. 2C). As latent infection may both represent an upcoming disease or an asymptomatic infection, we decided to exclude these data from further analyses, focusing only on healthy and diseased plants.

### Divergence in microbiome composition predicts disease outcome

**Fig. 3:**
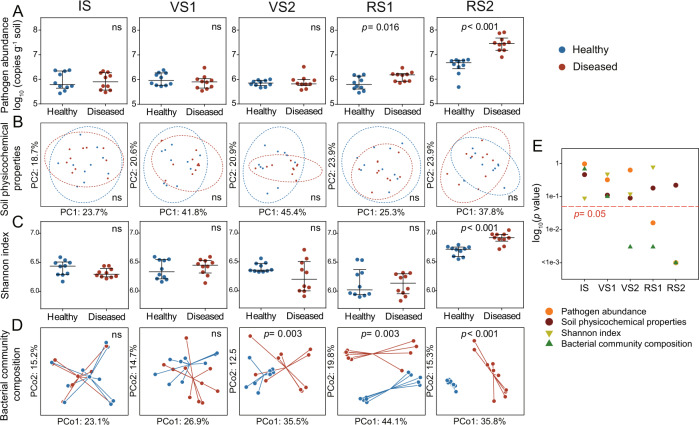
The initially uniform soil microbiome rapidly diverges into distinct states with implications for plant health. Dynamics of *R. solanacearum* abundance (**A**), soil physicochemical properties (**B**), Shannon index (**C**), and bacterial community composition **D** associated with plants classified at the end of the experiment into healthy (no to low pathogen density, no symptoms) and diseased (high pathogen density and clear wilt symptoms) plant individuals. Soil samples associated with ten plants of each disease outcome were randomly selected. IS initial stage, VS1 vegetative stage 1, VS2 vegetative stage 2, RS1 reproductive stage 1, RS2 reproductive stage 2. **E** Statistical significances (log-transformed *p* values) between the healthy and diseased plants with soil physicochemical properties, pathogen abundance, Shannon index, and bacterial community composition as response variables. Bacterial alpha and beta diversity (**C**, **D**) were estimated using the smallest sample depth (23, 639 reads). **A**, **C** pairwise comparisons (*p* values) between the two health states classified at the end of the experiment were conducted using the Student’s *t* test with pathogen density and Shannon index as response variables, respectively. **B** Physicochemical properties of rhizosphere soil (EC, pH, NH_4_^+^-N, NO_3_^−^-N, AP, TC, TN, DOC, DON, and CN ratio) were ordinated using principal component analysis (PCA). Ellipses indicate 95% confidence intervals around the clusters of healthy and disease plants. **D** Bacterial community composition was ordinated using principal coordinates analysis (PCoA) based on Bray–Curtis distances. Individual points are connected by half-lines radiated from the centroid. Statistical differences in soil physicochemical properties and bacterial community composition associated with the disease outcome (**B**, **D**) were determined by PERMANOVA. **E** The red dashed line indicates *p* = 0.05.

Similar to the disease incidence (Fig. 2B), the abundance of *R. solanacearum* remained low in healthy and diseased plants until abruptly increasing during the reproductive stage (Fig. 3A). The abundance of *Ralstonia solanacearum* was not significantly different in soils associated with the disease outcome until RS1, where a higher number of *R. solanacearum* was detected in the rhizosphere of diseased plants (1.3-fold, *p* = 0.016, Student’s *t* test), compared to that of healthy plants (Fig. 3A). Physicochemical properties of the rhizosphere soil exhibited successional patterns across plant developmental stages, however, these patterns were not linked to the disease outcome (*p* > 0.05, PERMANOVA; Fig. 3B, Fig. S3, and Table S4). This suggests that variations in soil physicochemical properties were not likely to influence the disease outcome. The rhizosphere bacterial communities showed significant clustering according to different plant developmental stages (Fig. S4; *p* < 0.001, PERMANOVA). We found the initial significant difference in bacterial community composition between healthy and diseased tomato plants at VS2 (Fig. 3D), 2 weeks early than any detectable changes in pathogen density (Fig. 3E). This occurs, even though, significant differences in community alpha diversity between healthy and diseased tomato were only observed at RS2 (*p* < 0.001, Student’s *t* test) (Fig. 3C). We further repeated the analyses of bacterial community composition at zero-radius OTU (zOTU) level. We found a strong correlation in patterns observed based on the OTU and zOTU methods (Fig. S5A), i.e., initially similar microbiome composition diverging into multiple configurations at VS2 (Fig. S5B).

### Discriminating taxa associated with disease development

**Fig. 4:**
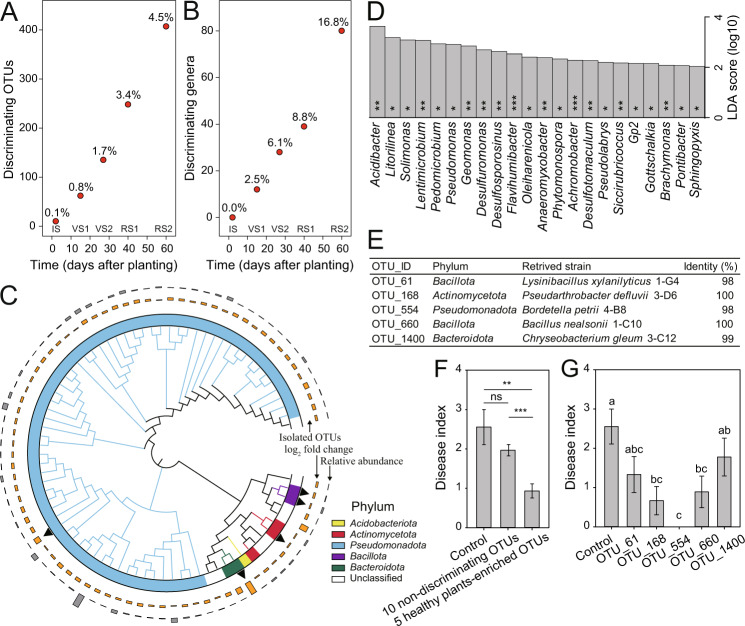
Taxa enriched in healthy plants when contrasting community states formed have potential disease suppression ability. The number of discriminating OTUs (**A**) and genera (**B**) (LDA score >2, *p* < 0.05) between healthy and disease plants. The percentages indicate the proportion of discriminating OTUs/genera in all OTUs/genera for each plant developmental stage. IS initial stage, VS1 vegetative stage 1, VS2 vegetative stage 2, RS1 reproductive stage 1, RS2 reproductive stage 2. **C** Phylogenetic tree of OTUs enriched in healthy plants at vegetative stage 2. Different strip colors indicate the phylum affiliation of OTUs enriched in healthy plants. The black triangles indicate the five isolated OTUs. These OTUs were obtained from the rhizosphere soils of tomato plants destructively collected at vegetative stage 2 (performed in an independent greenhouse experiment). All five OTUs were enriched in healthy plants at vegetative stage 2. The gray bars indicate the average relative abundance of each OTU in the soil associated with healthy and diseased plants, and the orange bars indicate the log_2_ fold change. **D** Genera enriched in healthy plants at vegetative stage 2 (*p < 0.05; **p < 0.01; ***p < 0.001). **E** Isolated OTUs enriched in healthy plants at vegetative stage 2. **F** Disease suppression ability of ten non-discriminating OTUs between healthy and diseased plants and five healthy-plant-enriched OTUs at vegetative stage 2 (*n* = 18). **p < 0.01; ***p < 0.001. **G** Disease suppression of the five healthy-plant-enriched OTUs at vegetative stage 2 (*n* = 18).

We identified discriminating taxa associated with disease outcomes for each plant developmental stage. We found that the number of these discriminating OTUs (0.1–4.5% of total OTU number) and discriminating genera (0–16.8% of total genus number) increased with plant development (Fig. 4A, B). These suggest that the divergence of the whole microbiome composition at VS2 might have been due to events that had already occurred previously. Thus, the distinct states of the microbiome composition may likely reflect initial divergences in community assembly. In particular, a large proportion (80.2%) of healthy-plant-enriched OTUs at VS2 was *Pseudomonadota* (Fig. 4C), while most of the diseased-plant-enriched OTUs were *Bactroidota* (20.4%), *Verrucomicrobiota* (18.5%), and unclassified bacteria (29.6%) (Table S5). Twenty-two genera were significantly enriched in healthy plants at VS2, including 12 *Pseudomonadota* members (*Acidibacter*, *Solimonas*, *Pedomicrobium*, *Pseudomonas*, *Geomonas*, *Desulfuromonas*, *Anaeromyxobacter*, *Achromobacter*, *Pseudolabrys*, *Siccirubricoccus*, *Brachymonas*, and *Sphingopyxis*) (Fig. 4D).

To test whether the healthy-plant-enriched rhizobacteria at VS2 contribute to disease suppression, we isolated bacteria from the rhizosphere soil at VS2 (27 dap). All the 137 isolates were matched to 31 OTUs, out of which five OTUs were associated with later healthy plants. These include *Lysinibacillus xylanilyticus*, *Pseudarthrobacter defluvii*, *Bordetella petrii*, *Bacillus nealsonii*, and *Chryseobacterium gleum* (Fig. 4C, E). We further carried out a greenhouse experiment by inoculating plants with the isolated strains to test their suppression against *R. solanacearum*. We found that the five discriminating OTUs/strains significantly reduced the disease index of bacterial wilt (*p* < 0.01, nonparametric Mann–Whitney test) compared to the control. We also included ten randomly selected non-discriminating OTUs/strains to explore the disease suppression ability of strains by random chance. Importantly, the five discriminating OTUs/strains showed a higher capacity of disease suppression than the ten randomly selected non-discriminating OTUs/strains (*p* < 0.001, nonparametric Mann–Whitney test) (Fig. 4F, Fig. S6, and Table S3). Specifically, these five strains reduced the disease index by 30.4–100%. The effects of OTU_168 (*P. defluvii*), OTU_554 (*B. petrii*), and OTU_660 (*B. nealsonii*) were significantly greater (*p* < 0.05, Student’s *t* test) than the control (Fig. 4G). Together, these data validate the disease suppression ability of some healthy-plant-enriched OTUs at VS2. Moreover, we found four of the five suppressive isolates (OTU_61, OTU_168, OTU_660, and OTU_1400) to antagonize the growth of *R. solanacearum* by conducting supernatant assay (Fig. S7 and Appendix S2). Worth mentioning, the isolate corresponding to OTU_554 had no evident effect on the growth of the pathogen. This suggests that OTU_554 might have reduced the incidence of bacterial wilt via indirect effects, for example, inducing plant immunity. Together, the combination of culture-independent and culture-dependent approaches demonstrated that microbiome cues can predict disease outcomes before the appearance of symptoms, and that specific members of the rhizosphere microbiome can be harnessed to reduce disease severity.

## Discussion

In this study, we addressed whether disease suppressiveness could emerge during microbiome assembly, without the need for the initial presence of a specific set of species or environmental conditions. We showed that an initially homogeneous bulk soil microbiomes (i.e., homogeneous species pool) can result in different rhizosphere microbiomes with direct implications for plant health/protection. In this case, these variations in community composition allowed us to predict the disease onset, earlier than noticeable changes in the pathogen density. This rapid divergence during the assembly process sheds new light on disease dynamics and provides new avenues for microbiome diagnostics and for managing microbiome-driven plant disease resistance.

This study contributes to answering the (at a first glance) erratic nature of disease outcomes [38]. We first used a meta-analysis to reveal that outbreaks of bacterial wilt disease consistently show a binary outcome with individuals either remaining healthy or diseased. Binary disease outcomes have long been reported as anecdotes across many studies. It is traditionally explained by the heterogeneity of biotic and abiotic stress exposure [39], pathogen pressure [38], or variation in the microbial species pool [40–42]. This study provides an alternative explanation to these previously reported constraints. Simply, the microbiome composition rapidly diverges into different states, out of an initially homogeneous bacterial species pool. Then, this variability is strongly linked to the binary disease outcome. This discovery was made possible by the use of a longitudinal study, an approach long used in human medicine but only applied to plant-microbiome studies recently. This approach allows for unraveling causalities between microbiome assembly and disease onset. This is particularly important as the use of marker species after disease onset, a procedure still often used in microbiome studies, will highlight species able to live in the sick environment dominated by the pathogen [16]. As such, blurring our ability to early detect the main taxa involved with pathogen suppression.

Pathogen density is widely used as a predictor/indicator of disease development [43, 44]. However, it is often late for disease control when high pathogen density is detected. Using our study as an example, *R. solanacearum* density significantly increased at the flowering stage when certain tomato plants had already shown wilt symptoms. Therefore, an early predictor is needed for the disease diagnosis prior to pathogen outbreaks to timely control the disease. Here, we found that the rhizosphere microbiome composition can predict the disease outcome even 2 weeks before any detectable changes in the pathogen abundance between the later healthy and diseased plants. This result indicates the potential disease suppression ability of the rhizosphere microbiome at the seedling stage. It also highlights the importance of optimizing seedling management to sustainably enhance plant health by focusing on the functioning of root-associated microbiomes. Additionally, the association between microbiome composition and disease outcomes may contribute to microbiome-based diagnostics where microbiome composition can be used to develop models on disease occurrence and severity. It is worth pointing out that we found diseased plants to be associated with higher rhizosphere bacterial community diversity than healthy plants. It is generally recognized that pathogen infection can reduce rhizosphere microbiome diversity [15, 17, 45]. However, plant diseases have also been found to increase or have no effects on rhizosphere microbiome diversity [7, 46, 47]. These discrepancies might occur due to differences in species pool across soils of different origins, the genetic background of the plant host, the nature/physiology/ecology of the pathogen, differences in soil management/agricultural practices, and/or environmental conditions [5, 48, 49].

Changes in the rhizosphere community composition rather than the overall species diversity can explain future plant health. At a more detailed level, we found that disease-suppressive microbiome states were associated with specific microbial taxa. The presence of discriminating taxa points to an over-proportional role of some species as drivers of microbiome suppressiveness [40]. In this study, we retrieved a range of bacterial isolates matching genetically these discriminating taxa. When inoculated in tomato plants, these strains provided suppression against the pathogen to a level higher than expected by chance, thus supporting their potential role as mediators of plant health. We acknowledge that additional research is needed to explain how these specific taxa operate and were enriched in some samples and whether the mechanisms involved in disease suppression are generalizable. Several discriminating taxa belonged to *Pseudomonadota*, a phylum associated with gut and rhizosphere health, pointing to a general pattern [50]. Other taxa belonged to bacterial groups previously reported to harbor a broad antipathogen potential, such as *Pseudomonas, Achromobacter*, *Brachymonas*, or *Sphingopyxis* [51–54]. However, we would like to state here that the goal of our isolation-inoculation approach here was merely to validate our findings rather than providing a comprehensive mechanistic screening of rhizosphere suppressive taxa. For instance, most of the healthy-plant-enriched *Pseudomonadota* could not be successfully retrieved by standard isolation procedures, and the five protective isolates reported here represent only a small fraction of the potential multiple mechanisms operating on disease suppression by the several healthy-plant-enriched taxa.

Our results showed that small and early differences in the rhizosphere microbiome assembling further influence plant health. As the dynamics of microbiome assembly show strong parallels between hosts, we anticipate that the results will be of direct relevance to explaining the dynamics of disease outbreaks. Besides, such principle can be used to design early microbiome-centered interventions in diverse ecosystems, such as human/animal gut or coral reefs [55–57]. While further studies are needed to establish the possible mechanisms leading to divergence in microbiome assembly in this study, this effect can be a result of microbiome intrinsic dynamics. For example, the root exudates secreted by growing roots of plant individuals reduced competitive pressures leading to stochastic processes in microbial interactions, which may further lead to changes in microbiome composition [58]. Recently, Matsumoto et al. [38] reported that rice of the same cultivar can shift to disease-resistant and susceptible phenotypes under the pressure of the same seed-borne pathogen due to the different disease resistance conferred by the seed microbiome. Our study suggests that the distinct state of the rhizosphere microbiome composition can also be a result of changes in the interactions between the seed microbiome and the soil microbiome. This study focused on rhizosphere bacteria as a primary level of protection against pathogen invasion. Due to the technical impossibility to sample endophytic communities in a nondestructive way, we did not include the endosphere in our study, even though endophytes might also contribute to asymptomatic infections under high pathogen density [59].

Bacterial wilt is commonly characterized by symptomless latent infection [4, 60], while the incidence of the latent infection is scarcely reported. Thus, further studies are needed to unravel the incidence of latent infection under different growth conditions. In this study, we omitted latent infections as they may lump together unrelated processes (i.e., delayed symptom onset and asymptomatic infection). We did nonetheless analyze their rhizosphere communities and found that the rhizosphere microbiome composition of latently infected plants was different from that of the healthy plants at VS2 (Table S6). This suggests that the establishment of latent infection was likely associated with the microbiome status. In addition, none of the five protective isolates in this study were enriched in the rhizosphere of the latently infected plants (Fig. S8), thus suggesting that lower relative abundances of these protective taxa might likely result in greater pathogen success.

## Conclusion

Plants growing in homogeneous conditions and in the presence of a soilborne pathogen often display a binary disease outcome. However, the underlying nature of this phenomenon remains still largely underexplored. Here, we show that the rhizosphere microbiome composition of individual plants developed towards significantly different states even before the outbreak of the pathogen abundance. Furthermore, the changes in the rhizosphere microbiome composition could predict whether plants remained healthy or became infected by the pathogen. While pathogen density can predict disease development, it is challenging to predict disease outcomes in homogenous soils having the same initial pathogen density. Our results highlight an opportunity for microbiome diagnostics of plant diseases by profiling the microbiome as in human disease. Importantly, healthy-plant-enriched taxa, when distinct community states formed, reduced disease incidence, suggesting the potential disease suppression ability of the healthy plant-associated microbiome. These taxa may be targeted as a viable alternative to promote disease suppression toward the progressive reduction of environmentally hazardous pesticides.

## Supplementary information

Supplementary information

External Databases S1

## Data Availability

All data needed to evaluate the conclusions in the paper are present in the paper and/or the Supplementary Materials. Raw sequencing sequences were deposited in the DDBJ SRA under the accession numbers PRJNA299538 and PRJNA806399. Additional data related to this paper may be requested from the corresponding author ZW (weizhong@njau.edu.cn).

## References

[1] Dean R, Van Kan JA, Pretorius ZA, Hammond-Kosack KE, Di Pietro A, Spanu PD (2012). The Top 10 fungal pathogens in molecular plant pathology. Mol Plant Pathol.

[2] Mansfield J, Genin S, Magori S, Citovsky V, Sriariyanum M, Ronald P (2012). Top 10 plant pathogenic bacteria in molecular plant pathology. Mol Plant Pathol.

[3] Campbell CL, Noe JP (1985). The spatial analysis of soilborne pathogens and root diseases. Annu Rev Phytopathol.

[4] Genin S, Denny TP (2012). Pathogenomics of the *Ralstonia solanacearum* species complex. Annu Rev Phytopathol.

[5] Kwak MJ, Kong HG, Choi K, Kwon SK, Song JY, Lee J (2018). Rhizosphere microbiome structure alters to enable wilt resistance in tomato. Nat Biotechnol.

[6] Wei Z, Gu Y, Friman V-P, Kowalchuk GA, Xu Y, Shen Q (2019). Initial soil microbiome composition and functioning predetermine future plant health. Sci Adv.

[7] Lee SM, Kong HG, Song GC, Ryu CM (2021). Disruption of *Firmicutes* and *Actinobacteria* abundance in tomato rhizosphere causes the incidence of bacterial wilt disease. ISME J.

[8] Berendsen RL, Pieterse CM, Bakker PA (2012). The rhizosphere microbiome and plant health. Trends Plant Sci.

[9] Hu J, Wei Z, Kowalchuk GA, Xu Y, Shen Q, Jousset A (2020). Rhizosphere microbiome functional diversity and pathogen invasion resistance build up during plant development. Environ Microbiol.

[10] Faust K, Lahti L, Gonze D, de Vos WM, Raes J (2015). Metagenomics meets time series analysis: unraveling microbial community dynamics. Curr Opin Microbiol.

[11] Fuentes-Chust C, Parolo C, Rosati G, Rivas L, Perez-Toralla K, Simon S (2021). The microbiome meets nanotechnology: opportunities and challenges in developing new diagnostic devices. Adv Mater.

[12] Schlaberg R (2020). Microbiome diagnostics. Clin Chem.

[13] Xiao Y, Yang C, Yu L, Tian F, Wu Y, Zhao J (2021). Human gut-derived *B. longum* subsp. *longum* strains protect against aging in a D-galactose-induced aging mouse model. Microbiome.

[14] Petrova MI, Lievens E, Malik S, Imholz N, Lebeer S (2015). *Lactobacillus* species as biomarkers and agents that can promote various aspects of vaginal health. Front Physiol.

[15] Wei Z, Hu J, Gu Y, Yin S, Xu Y, Jousset A (2018). *Ralstonia solanacearum* pathogen disrupts bacterial rhizosphere microbiome during an invasion. Soil Biol Biochem.

[16] Gu S, Wei Z, Shao Z, Friman V-P, Cao K, Yang T (2020). Competition for iron drives phytopathogen control by natural rhizosphere microbiomes. Nat Microbiol.

[17] Wei Z, Yang T, Friman V-P, Xu Y, Shen Q, Jousset A (2015). Trophic network architecture of root-associated bacterial communities determines pathogen invasion and plant health. Nat Commun.

[18] Li M, Pommier T, Yin Y, Wang J, Gu S, Jousset A (2022). Indirect reduction of *Ralstonia solanacearum* via pathogen helper inhibition. ISME J.

[19] Dubinkina V, Fridman Y, Pandey PP, Maslov S (2019). Multistability and regime shifts in microbial communities explained by competition for essential nutrients. Elife.

[20] Coyte KZ, Schluter J, Foster KR (2015). The ecology of the microbiome: networks, competition, and stability. Science.

[21] Garcia-Palacios P, Vandegehuchte ML, Shaw EA, Dam M, Post KH, Ramirez KS (2015). Are there links between responses of soil microbes and ecosystem functioning to elevated CO_2_, N deposition and warming? A global perspective. Glob Chang Biol.

[22] Chen Y, Yan F, Chai Y, Liu H, Kolter R, Losick R (2013). Biocontrol of tomato wilt disease by *Bacillus subtilis* isolates from natural environments depends on conserved genes mediating biofilm formation. Environ Microbiol.

[23] Elphinstone J, Hennessy J, Wilson J, Stead D (1996). Sensitivity of different methods for the detection of *Ralstonia solanacearum* in potato tuber extracts. EPPO Bull.

[24] Schonfeld J, Heuer H, van Elsas JD, Smalla K (2003). Specific and sensitive detection of *Ralstonia solanacearum* in soil on the basis of PCR amplification of *fliC* fragments. Appl Environ Microbiol.

[25] Wei Z, Yang X, Yin S, Shen Q, Ran W, Xu Y (2011). Efficacy of *Bacillus*-fortified organic fertiliser in controlling bacterial wilt of tomato in the field. Appl Soil Ecol.

[26] Cardenas E, Wu WM, Leigh MB, Carley J, Carroll S, Gentry T (2010). Significant association between sulfate-reducing bacteria and uranium-reducing microbial communities as revealed by a combined massively parallel sequencing-indicator species approach. Appl Environ Microbiol.

[27] Gu Y, Wei Z, Wang X, Friman V-P, Huang J, Wang X (2016). Pathogen invasion indirectly changes the composition of soil microbiome via shifts in root exudation profile. Biol Fertil Soils.

[28] Kozich JJ, Westcott SL, Baxter NT, Highlander SK, Schloss PD (2013). Development of a dual-index sequencing strategy and curation pipeline for analyzing amplicon sequence data on the MiSeq Illumina sequencing platform. Appl Environ Microbiol.

[29] Edgar RC (2013). UPARSE: highly accurate OTU sequences from microbial amplicon reads. Nat Methods.

[30] Edgar RC, Haas BJ, Clemente JC, Quince C, Knight R (2011). UCHIME improves sensitivity and speed of chimera detection. Bioinformatics.

[31] Edgar RC UNOISE2: improved error-correction for Illumina 16S and ITS amplicon sequencing. BioRxiv. 2016. 10.1101/081257.

[32] Wang Q, Garrity GM, Tiedje JM, Cole JR (2007). Naive Bayesian classifier for rapid assignment of rRNA sequences into the new bacterial taxonomy. Appl Environ Microbiol.

[33] Olsen SR, Cole CV, Watanabe FS, Dean L. Estimation of available phosphorus in soils by extraction with sodium bicarbonate. Circ no. 939. Washington, DC: United States Department of Agriculture; 1954.

[34] Heuer H, Krsek M, Baker P, Smalla K, Wellington E (1997). Analysis of actinomycete communities by specific amplification of genes encoding 16S rRNA and gel-electrophoretic separation in denaturing gradients. Appl Environ Microbiol.

[35] R Core Team. R: A language and environment for statistical computing. Vienna, Austria: R Foundation for Statistical Computing; 2013.

[36] Oksanen J, Kindt R, Legendre P, O’Hara B, Stevens MHH, Oksanen MJ (2007). The vegan package. Community Ecol package.

[37] Segata N, Izard J, Waldron L, Gevers D, Miropolsky L, Garrett WS (2011). Metagenomic biomarker discovery and explanation. Genome Biol.

[38] Matsumoto H, Fan X, Wang Y, Kusstatscher P, Duan J, Wu S (2021). Bacterial seed endophyte shapes disease resistance in rice. Nat Plants.

[39] Bardgett RD, Caruso T (2020). Soil microbial community responses to climate extremes: resistance, resilience and transitions to alternative states. Proc R Soc Lond Ser B..

[40] Mendes R, Kruijt M, de Bruijn I, Dekkers E, van der Voort M, Schneider JH (2011). Deciphering the rhizosphere microbiome for disease-suppressive bacteria. Science..

[41] Raaijmakers JM, Mazzola M (2016). Soil immune responses. Science..

[42] Gu Y, Wang X, Yang T, Friman VP, Geisen S, Wei Z (2020). Chemical structure predicts the effect of plant-derived low molecular weight compounds on soil microbiome structure and pathogen suppression. Funct Ecol.

[43] Burdon J, Chilvers G (1982). Host density as a factor in plant disease ecology. Annu Rev Phytopathol.

[44] Rosenfeld M, Gibson RL, McNamara S, Emerson J, Burns JL, Castile R (2001). Early pulmonary infection, inflammation, and clinical outcomes in infants with cystic fibrosis. Pediatr Pulmonol.

[45] Li J-G, Ren G-D, Jia Z-J, Dong Y-H (2014). Composition and activity of rhizosphere microbial communities associated with healthy and diseased greenhouse tomatoes. Plant Soil.

[46] Liu X, Zhang S, Jiang Q, Bai Y, Shen G, Li S (2016). Using community analysis to explore bacterial indicators for disease suppression of tobacco bacterial wilt. Sci Rep.

[47] Filion M, Hamelin RC, Bernier L, St-Arnaud M (2004). Molecular profiling of rhizosphere microbial communities associated with healthy and diseased black spruce (*Picea mariana*) seedlings grown in a nursery. Appl Environ Microbiol.

[48] Gu Y, Dong K, Geisen S, Yang W, Yan Y, Gu D (2020). The effect of microbial inoculant origin on the rhizosphere bacterial community composition and plant growth-promotion. Plant Soil.

[49] Jiang G, Wang N, Zhang Y, Wang Z, Zhang Y, Yu J (2021). The relative importance of soil moisture in predicting bacterial wilt disease occurrence. Soil Ecol Lett.

[50] Mendes R, Raaijmakers JM (2015). Cross-kingdom similarities in microbiome functions. ISME J.

[51] Dhaouadi S, Rouissi W, Mougou-Hamdane A, Nasraoui B (2018). Evaluation of biocontrol potential of *Achromobacter xylosoxidans* against *Fusarium* wilt of melon. Eur J Plant Pathol.

[52] Halet D, Defoirdt T, Van Damme P, Vervaeren H, Forrez I, Van de Wiele T (2007). Poly-beta-hydroxybutyrate-accumulating bacteria protect gnotobiotic *Artemia franciscana* from pathogenic *Vibrio campbellii*. FEMS Microbiol Ecol.

[53] Fujiwara K, Iida Y, Someya N, Takano M, Ohnishi J, Terami F (2016). Emergence of antagonism against the pathogenic fungus *Fusarium oxysporum* by interplay among non-antagonistic bacteria in a hydroponics using multiple parallel mineralization. J Phytopathol.

[54] Garbeva P, Silby MW, Raaijmakers JM, Levy SB, de Boer W (2011). Transcriptional and antagonistic responses of *Pseudomonas fluorescens* Pf0-1 to phylogenetically different bacterial competitors. ISME J..

[55] Sato Y, Willis BL, Bourne DG (2010). Successional changes in bacterial communities during the development of black band disease on the reef coral, Montipora hispida. ISME J.

[56] Glasl B, Herndl GJ, Frade PR (2016). The microbiome of coral surface mucus has a key role in mediating holobiont health and survival upon disturbance. ISME J.

[57] Burns AR, Stephens WZ, Stagaman K, Wong S, Rawls JF, Guillemin K (2016). Contribution of neutral processes to the assembly of gut microbial communities in the zebrafish over host development. ISME J.

[58] Badri DV, Chaparro JM, Zhang R, Shen Q, Vivanco JM (2013). Application of natural blends of phytochemicals derived from the root exudates of *Arabidopsis* to the soil reveal that phenolic-related compounds predominantly modulate the soil microbiome. J Biol Chem.

[59] Afzal I, Shinwari ZK, Sikandar S, Shahzad S (2019). Plant beneficial endophytic bacteria: mechanisms, diversity, host range and genetic determinants. Microbiol Res.

[60] Swanson JK, Montes L, Mejia L, Allen C (2007). Detection of Latent Infections of *Ralstonia solanacearum* Race 3 Biovar 2 in geranium. Plant Dis.

