# Supplementary material for: Small changes in rhizosphere microbiome composition predict disease outcomes earlier than pathogen density variations
Source: ISME J. 2022 Jul 22;16(10):2448–56. doi: 10.1038/s41396-022-01290-z (PMC9478146; doi:10.1038/s41396-022-01290-z)
Supplement: Supplementary file 1 — Supplementary information [file 41396_2022_1290_MOESM1_ESM.docx]

**Supplementary Information**

**This file includes:**

**Appendix S1-S2**

**Table S1 to S6**

**Fig. S1 to S8**

**Supplementary Materials and Methods**

**Appendix S1: Mesocosm system ("rhizobox")**

The rhizobox consisted of a three-layer cylinder (height, 136 mm; diameter, 110 mm) (Fig. S1A-S1B). The outer layer cylinder (4 mm metal mesh) supported the rhizobox and the inner layer (50 µm nylon mesh; root compartment) restricted plant roots. Eighteen soil-filled bags of nylon mesh (height, 136 mm; width, 18-21 mm, thickness, 1-2 mm; mesh size, 150 µm) were arranged between the outer and the inner layers of the rhizobox to create the middle sampling layer. Each middle-layer nylon bags was filled with 4 g of homogenized tomato soil collected in Qilin (< 2 mm), sprayed with sterile distilled water, gently squeezed to 1- to 2-mm thickness, and arranged between the outer and the inner layers of the rhizobox. The soils in the nylon mesh bags (*i.e.*, rhizosphere soils) can thus be affected by the surrounding soil outside the rhizobox and by the growth of plant roots in the root compartment (Fig. 1C). Each middle-layer nylon bag could be removed individually and three of the 18 middle-layer nylon bags were randomly collected at each sampling time.

**Supplementary Materials and Methods**

**Appendix S2: Effects of isolated rhizobacteria on pathogen growth**

We tested the direct effects of the five healthy-plant-enriched OTUs at vegetative stage 2 on the growth of *Ralstonia solanacearum* strain QL-Rs1115 *in vitro* by conducting supernatant experiment. Briefly, each of the five strains were cultivated in NB medium (glucose 10.0 g L^-1^, tryptone 5.0 g L^-1^, yeast extract 0.5 g L^-1^, beef extract 3.0 g L^-1^, pH 7.0) for 48 h (30 °C, 170 rpm). Bacterial monocultures were then passed through a 0.22 µm filter to remove living cells. Twenty microliters of sterile supernatant of each strain and 2 µl overnight culture of the pathogen (adjusted to OD600 = 0.5 in 0.9% sterile NaCl) were added into 178 µl of fresh 20% NB medium (to better reflect the effect of the supernatant) in 96-well plate. Twenty microliters of 0.9% sterile NaCl instead of the bacterial supernatant was included as a control. Each treatment consisted of three replicates. The 96-well plates were incubated for 24 h at 30°C with shaking (170 rpm) before measuring OD_600_ using a SpectraMax M5 plate reader. The effect of rhizobacteria on the growth of pathogen was defined as the percentage of reduction in pathogen growth by the supernatant compared to the control.

**Table S1 Primers used for PCR and quantitative PCR in this study.**

| Gene | Primer | Primer sequence (5'-3') | Thermal profile | Reference |
| --- | --- | --- | --- | --- |
| SSU rRNA | F27-R1492 | F - AGAGTTTGATCATGGCTCAG | 95°C-5 min; 30 cycle of 94°C-30 s, 58°C-30 s, and 72°C-1 min 30 s; 72°C-10 min | Heuer *et al*. 1997 |
|  |  | R -TACGGTTACCTTGTTACGACTT |  |  |
| SSU rRNA V4 region | 563F-802R | F - AYTGGGYDTAAAGVG | 95°C-2 min; 30 cycle of 95°C-30 s, 55°C-30 s, and 72°C-30 s; 72°C-5 min | Cardenas *et al*. 2010 |
|  |  | R - TACNVGGGTATCTAATCC |  |  |
| *fliC* | Rsol_*fliC* | F - GAACGCCAACGGTGCGAACT | 95°C-30 s; 40 cycle of 95°C-5 s, 60°C-34 s, and 72°C-30 s; 95°C-15 s, 60°C-1 min, 95°C-15 s | Schonfeld *et al*. 2003 |
|  |  | R - GGCGGCCTTCAGGGAGGTC |  |  |

**Table S2 Taxa of all the isolated bacteria.**

| Phylum | Genus | Strain number |
| --- | --- | --- |
| *Actinomycetota* |  | 22 |
|  | *Arthrobacter* | 12 |
|  | *Curtobacterium* | 1 |
|  | *Microbacterium* | 7 |
|  | *Rhodococcus* | 2 |
| *Bacteroidota* |  | 18 |
|  | *Chryseobacterium* | 5 |
|  | *Flavobacterium* | 9 |
|  | *Sphingobacterium* | 4 |
| *Bacillota* |  | 19 |
|  | *Bacillus* | 14 |
|  | *Fictibacillus* | 1 |
|  | *Lysinibacillus* | 2 |
|  | *Paenibacillus* | 2 |
| *Pseudomonadota* |  | 78 |
|  | *Acinetobacter* | 12 |
|  | *Bordetella* | 1 |
|  | *Comamonas* | 1 |
|  | *Cupriavidus* | 1 |
|  | *Delftia* | 1 |
|  | *Ensifer* | 1 |
|  | *Enterobacter* | 19 |
|  | *Klebsiella* | 5 |
|  | *Kosakonia* | 2 |
|  | *Ochrobactrum* | 3 |
|  | *Providencia* | 2 |
|  | *Pseudomonas* | 8 |
|  | *Sphingobium* | 2 |
|  | *Stenotrophomonas* | 18 |
|  | *Variovorax* | 1 |
|  | *Atlantibacter* | 1 |

**Table S3 Ten randomly selected non-discriminating isolates/OTUs used for disease control experiment.**

| OTU_ID | Phylum | Retrived strain | Identity (%) |
| --- | --- | --- | --- |
| OTU_57 | *Bacillota* | *Bacillus bataviensis* 6-E1 | 100 |
| OTU_452 | *Bacillota* | *Bacillus pumilus* 1-A5 | 100 |
| OTU_873 | *Pseudomonadota* | *Pseudomonas fluorescens* 1-B2 | 99 |
| OTU_1270 | *Bacillota* | *Falsibacillus pallidus* 4-H6 | 99 |
| OTU_1680 | *Actinobacteria* | *Microbacterium paraoxydans* 3-A6 | 98 |
| OTU_1748 | *Pseudomonadota* | *Acinetobacter junii* 3-D3 | 99 |
| OTU_1789 | *Pseudomonadota* | *Atlantibacter hermannii* 5-D2 | 98 |
| OTU_2745 | *Pseudomonadota* | *Acinetobacter calcoaceticus* 3-B1 | 97 |
| OTU_2910 | *Bacteroidota* | *Chryseobacterium taiwanense* 2-E5 | 97 |
| OTU_5665 | *Bacillota* | *Bacillus megaterium* 1-F3 | 100 |

**Table S4 Physicochemical properties of rhizosphere soil at different plant developmental stage.** Results show Mean ± SD. EC: electrical conductivity; DOC and DON denote dissolved organic carbon and dissolved organic nitrogen, respectively; NH_4_^+^-N and NO_3_^-^-N denote ammonium and nitrate nitrogen, respectively; AP, TN and TC denote available phosphate, total nitrogen and total carbon respectively. IS, initial stage; VS1, vegetative stage 1; VS2, vegetative stage 2; RS1, reproductive stage 1; RS2, reproductive stage 2.

| Soil properties | IS | VS1 | VS2 | RS1 | RS2 |
| --- | --- | --- | --- | --- | --- |
| DOC (mg kg^-1^) | 170.97 ± 5.25 | 180.01 ± 38.97 | 152.97 ± 29.59 | 178.75 ± 18.81 | 298.46 ± 47.91 |
| DON (mg kg^-1^) | 226.92 ± 6.59 | 209.17 ± 30.14 | 177.95 ± 23.13 | 137.10 ± 30.40 | 21.76 ± 4.59 |
| pH | 5.35 ± 0.08 | 5.44 ± 0.10 | 5.63 ± 0.05 | 5.60 ± 0.20 | 5.85 ± 0.46 |
| EC (μs cm^-1^) | 272.19 ± 7.46 | 249.65 ± 53.54 | 249.15 ± 43.43 | 174.40 ± 24.83 | 43.50 ± 6.44 |
| TN (%) | 0.67 ± 0.02 | 0.64 ± 0.03 | 0.61 ± 0.04 | 0.62 ± 0.03 | 0.58 ± 0.03 |
| TC (%) | 1.97 ± 0.06 | 1.97 ± 0.07 | 1.92 ± 0.05 | 1.97 ± 0.06 | 1.98 ± 0.05 |
| NH_4_^+^-N (mg kg^-1^) | 24.16 ± 0.80 | 23.80 ± 1.52 | 21.88 ± 0.59 | 20.83 ± 0.41 | 19.41 ± 0.67 |
| NO_3_^-^-N (mg kg^-1^) | 246.12 ± 7.20 | 236.68 ± 7.75 | 228.24 ± 8.94 | 194.85 ± 29.48 | 31.36 ± 10.44 |
| AP (mg kg^-1^) | 153.43 ± 4.65 | 141.52 ± 4.44 | 132.09 ± 6.22 | 136.17 ± 7.97 | 128.98 ± 4.18 |
| CN Ratio | 2.93 ± 0.10 | 3.07 ± 0.21 | 3.16 ± 0.22 | 3.18 ± 0.11 | 3.40 ± 0.16 |

**Table S5 List of OTUs enriched in healthy and diseased plants at vegetative stage 2.** log_2_ FC, log_2_ fold change.

| OTU_ID | Class | Phylum | LDA scroe | *p* value | log2 FC |
| --- | --- | --- | --- | --- | --- |
| OTU_1 | Diseased | *Bacteroidota* | 4.05 | 0.0191 | 0.84 |
| OTU_3 | Diseased | *Acidobacteriota* | 3.39 | 0.0041 | 0.39 |
| OTU_13 | Diseased | *Bacteroidota* | 3.25 | 0.0073 | 0.68 |
| OTU_14 | Diseased | *Verrucomicrobiota* | 3.28 | 0.0015 | 0.82 |
| OTU_19 | Diseased | *Bacteroidota* | 3.03 | 0.0493 | 0.55 |
| OTU_31 | Diseased | *Bacteroidota* | 2.82 | 0.0413 | 0.96 |
| OTU_36 | Diseased | Unclassified | 2.86 | 0.0234 | 0.68 |
| OTU_38 | Diseased | *Verrucomicrobiota* | 3.06 | 0.0008 | 1.02 |
| OTU_40 | Diseased | *Acidobacteriota* | 3.18 | 0.0311 | 0.54 |
| OTU_51 | Diseased | *Bacteroidota* | 3.10 | 0.0156 | 0.85 |
| OTU_55 | Diseased | Unclassified | 3.14 | 0.0036 | 1.04 |
| OTU_62 | Diseased | *Verrucomicrobiota* | 2.78 | 0.0340 | 0.96 |
| OTU_66 | Diseased | *Verrucomicrobiota* | 2.64 | 0.0072 | 0.86 |
| OTU_71 | Diseased | Unclassified | 2.26 | 0.0024 | 1.95 |
| OTU_72 | Diseased | *Bacteroidota* | 2.69 | 0.0373 | 0.75 |
| OTU_81 | Diseased | *Gemmatimonadota* | 2.69 | 0.0493 | 0.86 |
| OTU_85 | Diseased | *Verrucomicrobiota* | 2.56 | 0.0232 | 0.67 |
| OTU_87 | Diseased | Unclassified | 2.45 | 0.0140 | 0.55 |
| OTU_88 | Diseased | *Parcubacteria* | 2.66 | 0.0082 | 0.68 |
| OTU_92 | Diseased | Unclassified | 2.38 | 0.0446 | 0.62 |
| OTU_112 | Diseased | *Verrucomicrobiota* | 2.67 | 0.0411 | 0.92 |
| OTU_125 | Diseased | *Bacteroidota* | 2.58 | 0.0155 | 0.98 |
| OTU_127 | Diseased | Unclassified | 2.40 | 0.0031 | 0.77 |
| OTU_137 | Diseased | Unclassified | 2.50 | 0.0072 | 0.74 |
| OTU_151 | Diseased | *Pseudomonadota* | 2.70 | 0.0342 | 0.66 |
| OTU_153 | Diseased | Unclassified | 2.68 | 0.0081 | 0.84 |
| OTU_161 | Diseased | *Gemmatimonadota* | 2.37 | 0.0123 | 0.77 |
| OTU_188 | Diseased | *Chlamydiota* | 2.11 | 0.0095 | 0.52 |
| OTU_210 | Diseased | Unclassified | 2.21 | 0.0336 | 0.67 |
| OTU_229 | Diseased | Unclassified | 2.15 | 0.0184 | 0.53 |
| OTU_230 | Diseased | *Bacteroidota* | 2.07 | 0.0406 | 0.68 |
| OTU_241 | Diseased | Unclassified | 2.20 | 0.0306 | 0.54 |
| OTU_264 | Diseased | *Pseudomonadota* | 2.23 | 0.0080 | 1.48 |
| OTU_266 | Diseased | *Parcubacteria* | 2.37 | 0.0030 | 1.36 |
| OTU_269 | Diseased | *Gemmatimonadota* | 2.43 | 0.0097 | 1.22 |
| OTU_272 | Diseased | Unclassified | 2.09 | 0.0334 | 0.88 |
| OTU_299 | Diseased | Unclassified | 2.19 | 0.0231 | 0.81 |
| OTU_313 | Diseased | *Bacteroidota* | 2.07 | 0.0337 | 1.08 |
| OTU_334 | Diseased | *Bacteroidota* | 2.14 | 0.0395 | 0.62 |
| OTU_392 | Diseased | *Acidobacteriota* | 2.10 | 0.0322 | 0.69 |
| OTU_419 | Diseased | *Verrucomicrobiota* | 2.10 | 0.0371 | 0.66 |
| OTU_422 | Diseased | *Acidobacteriota* | 2.20 | 0.0019 | 0.77 |
| OTU_457 | Diseased | Unclassified | 2.12 | 0.0185 | 1.03 |
| OTU_474 | Diseased | *Pseudomonadota* | 2.24 | 0.0137 | 0.92 |
| OTU_619 | Diseased | *Pseudomonadota* | 2.19 | 0.0302 | 1.12 |
| OTU_631 | Diseased | Unclassified | 2.08 | 0.0078 | 1.29 |
| OTU_634 | Diseased | *Pseudomonadota* | 2.10 | 0.0164 | 1.99 |
| OTU_3099 | Diseased | *Parcubacteria* | 2.48 | 0.0232 | 0.72 |
| OTU_5375 | Diseased | *Verrucomicrobiota* | 2.34 | 0.0153 | 0.77 |
| OTU_5700 | Diseased | *Verrucomicrobiota* | 2.10 | 0.0422 | 1.05 |
| OTU_6311 | Diseased | Unclassified | 2.45 | 0.0310 | 1.19 |
| OTU_10397 | Diseased | *Gemmatimonadota* | 2.88 | 0.0125 | 1.10 |
| OTU_10472 | Diseased | *Bacteroidota* | 3.25 | 0.0102 | 0.79 |
| OTU_10807 | Diseased | *Verrucomicrobiota* | 2.90 | 0.0010 | 0.81 |
| OTU_4 | Healthy | *Pseudomonadota* | 3.13 | 0.0081 | 0.67 |
| OTU_6 | Healthy | *Pseudomonadota* | 3.86 | 0.0025 | 1.03 |
| OTU_8 | Healthy | *Pseudomonadota* | 2.94 | 0.0342 | 0.26 |
| OTU_11 | Healthy | *Pseudomonadota* | 2.96 | 0.0191 | 0.37 |
| OTU_15 | Healthy | *Pseudomonadota* | 3.02 | 0.0340 | 0.85 |
| OTU_20 | Healthy | *Pseudomonadota* | 3.33 | 0.0017 | 0.73 |
| OTU_25 | Healthy | *Pseudomonadota* | 3.05 | 0.0413 | 0.57 |
| OTU_29 | Healthy | *Pseudomonadota* | 3.09 | 0.0015 | 0.89 |
| OTU_35 | Healthy | *Pseudomonadota* | 2.95 | 0.0140 | 0.67 |
| OTU_48 | Healthy | *Pseudomonadota* | 3.37 | 0.0113 | 1.28 |
| OTU_50 | Healthy | *Pseudomonadota* | 3.13 | 0.0012 | 1.13 |
| OTU_61 | Healthy | *Bacillota* | 2.81 | 0.0312 | 0.50 |
| OTU_69 | Healthy | *Pseudomonadota* | 2.66 | 0.0413 | 0.28 |
| OTU_73 | Healthy | *Pseudomonadota* | 2.69 | 0.0011 | 0.77 |
| OTU_75 | Healthy | *Actinomycetota* | 2.90 | 0.0019 | 0.81 |
| OTU_82 | Healthy | *Pseudomonadota* | 2.87 | 0.0007 | 0.86 |
| OTU_91 | Healthy | *Pseudomonadota* | 2.64 | 0.0101 | 1.14 |
| OTU_98 | Healthy | Unclassified | 2.86 | 0.0002 | 0.94 |
| OTU_103 | Healthy | *Pseudomonadota* | 2.61 | 0.0081 | 0.78 |
| OTU_115 | Healthy | *Pseudomonadota* | 2.79 | 0.0126 | 0.63 |
| OTU_121 | Healthy | *Pseudomonadota* | 2.25 | 0.0281 | 0.22 |
| OTU_130 | Healthy | *Pseudomonadota* | 2.76 | 0.0015 | 1.17 |
| OTU_168 | Healthy | *Actinomycetota* | 2.16 | 0.0410 | 0.48 |
| OTU_170 | Healthy | *Pseudomonadota* | 2.27 | 0.0342 | 0.43 |
| OTU_182 | Healthy | *Pseudomonadota* | 2.01 | 0.0039 | 1.07 |
| OTU_192 | Healthy | *Pseudomonadota* | 2.38 | 0.0209 | 0.74 |
| OTU_199 | Healthy | *Pseudomonadota* | 2.39 | 0.0045 | 0.49 |
| OTU_208 | Healthy | Unclassified | 2.19 | 0.0486 | 0.45 |
| OTU_211 | Healthy | *Pseudomonadota* | 2.56 | 0.0010 | 1.43 |
| OTU_244 | Healthy | *Bacteroidota* | 2.14 | 0.0134 | 2.22 |
| OTU_270 | Healthy | *Pseudomonadota* | 2.32 | 0.0080 | 0.83 |
| OTU_282 | Healthy | *Pseudomonadota* | 2.52 | 0.0051 | 0.78 |
| OTU_293 | Healthy | Unclassified | 2.48 | 0.0001 | 2.84 |
| OTU_297 | Healthy | *Pseudomonadota* | 2.08 | 0.0371 | 0.67 |
| OTU_305 | Healthy | *Pseudomonadota* | 2.31 | 0.0490 | 0.72 |
| OTU_310 | Healthy | Unclassified | 2.03 | 0.0107 | 0.78 |
| OTU_312 | Healthy | *Pseudomonadota* | 2.20 | 0.0123 | 0.87 |
| OTU_316 | Healthy | *Pseudomonadota* | 2.04 | 0.0254 | 0.83 |
| OTU_324 | Healthy | *Pseudomonadota* | 2.13 | 0.0208 | 0.48 |
| OTU_347 | Healthy | *Pseudomonadota* | 2.05 | 0.0094 | 0.60 |
| OTU_354 | Healthy | *Pseudomonadota* | 2.41 | 0.0072 | 0.95 |
| OTU_358 | Healthy | *Pseudomonadota* | 2.37 | 0.0090 | 0.81 |
| OTU_362 | Healthy | *Pseudomonadota* | 2.38 | 0.0063 | 1.17 |
| OTU_369 | Healthy | *Pseudomonadota* | 2.32 | 0.0018 | 0.76 |
| OTU_461 | Healthy | Unclassified | 2.04 | 0.0246 | 1.00 |
| OTU_497 | Healthy | *Pseudomonadota* | 2.07 | 0.0069 | 0.72 |
| OTU_506 | Healthy | *Pseudomonadota* | 2.10 | 0.0254 | 1.04 |
| OTU_514 | Healthy | *Pseudomonadota* | 2.04 | 0.0049 | 1.06 |
| OTU_530 | Healthy | *Pseudomonadota* | 2.17 | 0.0063 | 1.07 |
| OTU_549 | Healthy | *Pseudomonadota* | 2.25 | 0.0491 | 0.49 |
| OTU_554 | Healthy | *Pseudomonadota* | 2.32 | 0.0002 | 2.16 |
| OTU_564 | Healthy | *Pseudomonadota* | 2.01 | 0.0075 | 1.11 |
| OTU_660 | Healthy | *Bacillota* | 2.42 | 0.0188 | 0.76 |
| OTU_679 | Healthy | *Pseudomonadota* | 2.07 | 0.0057 | 1.66 |
| OTU_700 | Healthy | *Pseudomonadota* | 2.07 | 0.0064 | 0.97 |
| OTU_707 | Healthy | *Pseudomonadota* | 2.16 | 0.0031 | 1.09 |
| OTU_716 | Healthy | Unclassified | 2.21 | 0.0099 | 0.83 |
| OTU_742 | Healthy | Unclassified | 2.10 | 0.0053 | 1.25 |
| OTU_925 | Healthy | *Pseudomonadota* | 2.30 | 0.0031 | 1.18 |
| OTU_1010 | Healthy | *Pseudomonadota* | 2.07 | 0.0125 | 0.96 |
| OTU_1167 | Healthy | *Pseudomonadota* | 2.06 | 0.0277 | 0.66 |
| OTU_1294 | Healthy | *Pseudomonadota* | 2.35 | 0.0007 | 1.01 |
| OTU_1303 | Healthy | *Pseudomonadota* | 2.38 | 0.0089 | 0.98 |
| OTU_1400 | Healthy | *Bacteroidota* | 2.07 | 0.0001 | 4.74 |
| OTU_1601 | Healthy | *Acidobacteriota* | 2.00 | 0.0485 | 0.42 |
| OTU_1844 | Healthy | *Pseudomonadota* | 2.86 | 0.0015 | 1.43 |
| OTU_2650 | Healthy | *Pseudomonadota* | 2.04 | 0.0131 | 1.06 |
| OTU_2988 | Healthy | *Actinomycetota* | 2.69 | 0.0211 | 0.42 |
| OTU_4413 | Healthy | *Pseudomonadota* | 3.12 | 0.0172 | 0.90 |
| OTU_5180 | Healthy | *Pseudomonadota* | 2.59 | 0.0006 | 1.02 |
| OTU_5561 | Healthy | *Pseudomonadota* | 2.64 | 0.0021 | 0.89 |
| OTU_5913 | Healthy | *Pseudomonadota* | 2.16 | 0.0411 | 0.69 |
| OTU_6721 | Healthy | *Pseudomonadota* | 2.32 | 0.0021 | 1.16 |
| OTU_6956 | Healthy | *Pseudomonadota* | 2.02 | 0.0372 | 0.71 |
| OTU_7679 | Healthy | *Pseudomonadota* | 2.95 | 0.0126 | 1.17 |
| OTU_7765 | Healthy | *Pseudomonadota* | 2.22 | 0.0137 | 0.55 |
| OTU_8216 | Healthy | *Pseudomonadota* | 2.44 | 0.0206 | 0.90 |
| OTU_10688 | Healthy | *Pseudomonadota* | 2.30 | 0.0408 | 0.46 |
| OTU_10988 | Healthy | *Pseudomonadota* | 2.26 | 0.0338 | 0.66 |
| OTU_11284 | Healthy | Unclassified | 2.57 | 0.0490 | 0.48 |
| OTU_12353 | Healthy | *Pseudomonadota* | 2.27 | 0.0028 | 1.17 |

**Table S6 Statistical significance (*p* value) of soil bacterial community composition of future healthy, latently infected, and diseased plants (PERMANOVA, n =10)*.*** H, L, and D indicate healthy, latently infected, and diseased plants, respectively. Significant variations are highlighted in bold.

| Response | Disease outcome | Initial stage | Vegetative stage 1 | Vegetative stage 2 | Reproductive stage 1 | Reproductive stage 2 |
| --- | --- | --- | --- | --- | --- | --- |
| Microbiome | H-L-D | 0.985 | 0.471 | **0.017** | **< 0.001** | **< 0.001** |
|  | H-D | — | — | **0.009** | **0.009** | **< 0.001** |
|  | H-L | — | — | **0.039** | **0.030** | **< 0.001** |
|  | L-D | — | — | 0.419 | **0.002** | **< 0.001** |


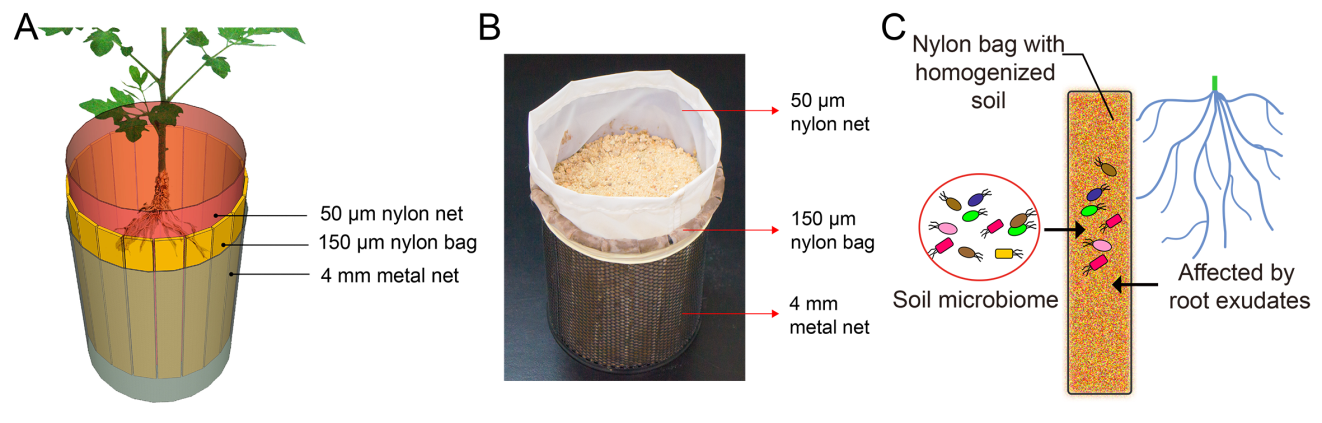


**Fig. S1 Soil collection device used in this study.** Schematic drawing (**A**) and real picture (**B**) of real picture of the soil collection device (Photo credit: Yian Gu, Nanjing Agricultural University). The soil in the nylon mesh bags of the middle layer was in close contact with plant roots and root exudates and was used as a proxy of rhizosphere bacterial community (**C**). Soil collection device that allows for nondestructive repeated sampling of individual plants at different growth stages was modified from Wei et al. (2019).


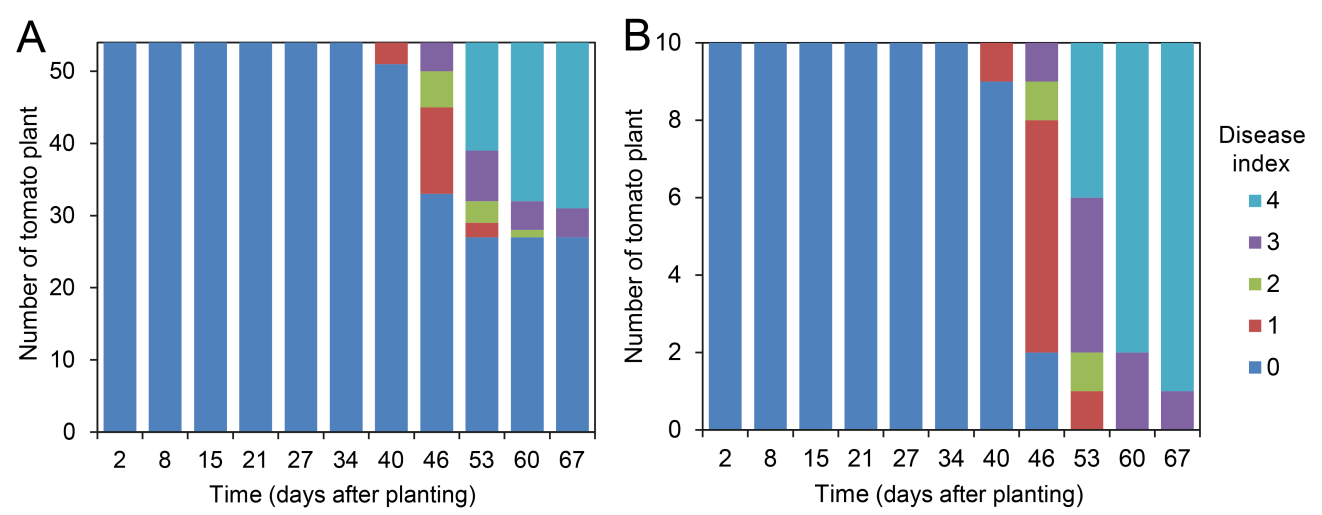


**Fig. S2 Dynamics of disease indexes of all the 54 plants (A) and ten randomly selected diseased plants (B).** Wilting symptoms were graded from 0 to 4.

**
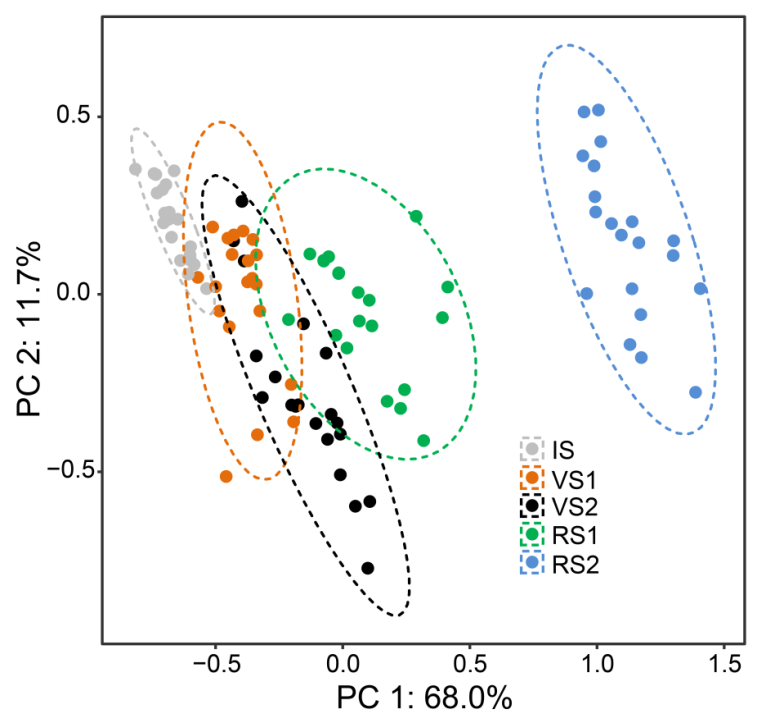
**

**Fig. S3 Principal component analysis (PCA) ordinations showing the variation in physicochemical properties of rhizosphere soil (EC, pH, NH_4_^+^-N, NO_3_^-^-N, AP, TC, TN, DOC, DON and CN ratio) dispersion by plant developmental stage.** Ellipses indicate 95% confidence interval around the clusters of different plant developmental stage. IS, initial stage; VS1, vegetative stage 1; VS2, vegetative stage 2; RS1, reproductive stage 1; RS2, reproductive stage 2.


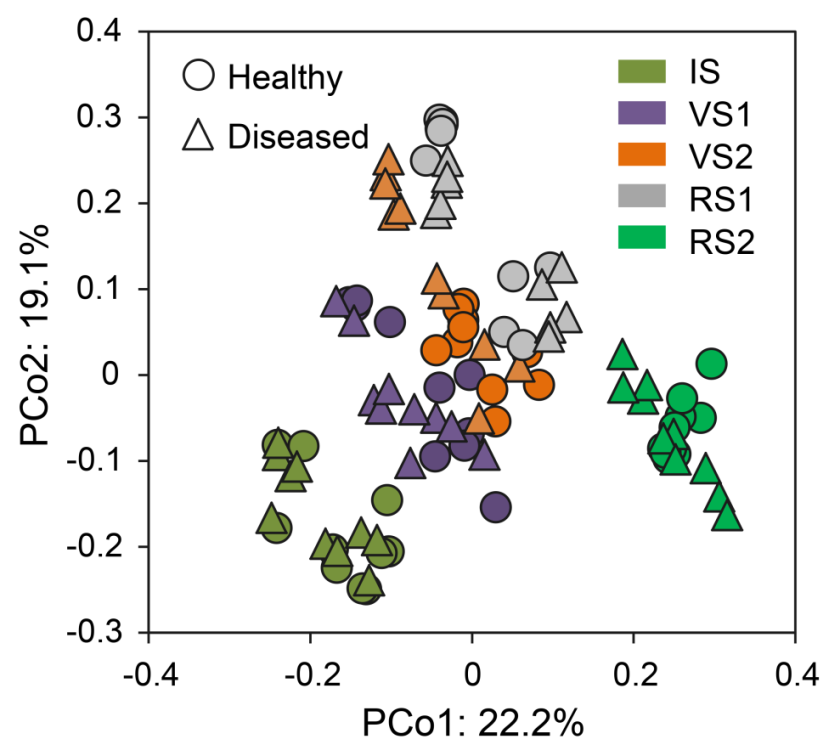


**Fig. S4 Variation in bacterial community composition dispersion by plant developmental stages and health states.** Bacterial community composition was ordinated using principal coordinates analysis (PCoA) based on Bray-curtis distance metric. IS, initial stage; VS1, vegetative stage 1; VS2, vegetative stage 2; RS1, reproductive stage 1; RS2, reproductive stage 2.


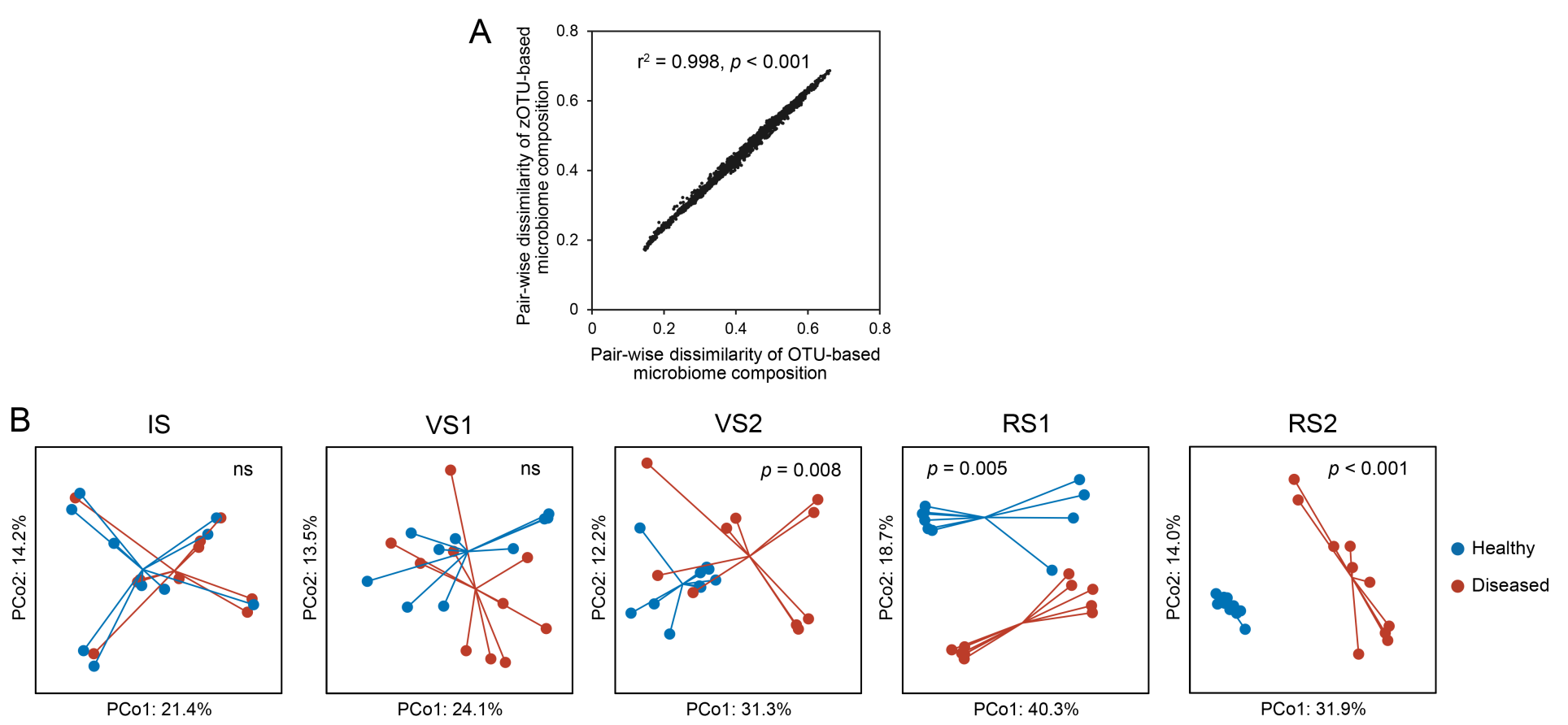


**Fig. S5 Correlational analysis between community dissimilarity patterns (Bray-Curtis) based on OTU and zOTU methods (A), and dynamics of bacterial community composition at zOTU level associated with plants classified at the end of the experiment into healthy and diseased plant individuals (B).** In panel A, pair-wise dissimilarity of bacterial community composition was calculated based on Bray-curtis distance metric. In panel B, bacterial community composition was ordinated using principal coordinates analysis (PCoA) based on Bray-curtis distance metric. Individual points are connected by half-lines radiated from the centroid. Statistical differences in the bacterial community composition associated with the disease outcome were determined by PERMANOVA test. IS, initial stage; VS1, vegetative stage 1; VS2, vegetative stage 2; RS1, reproductive stage 1; RS2, reproductive stage 2.


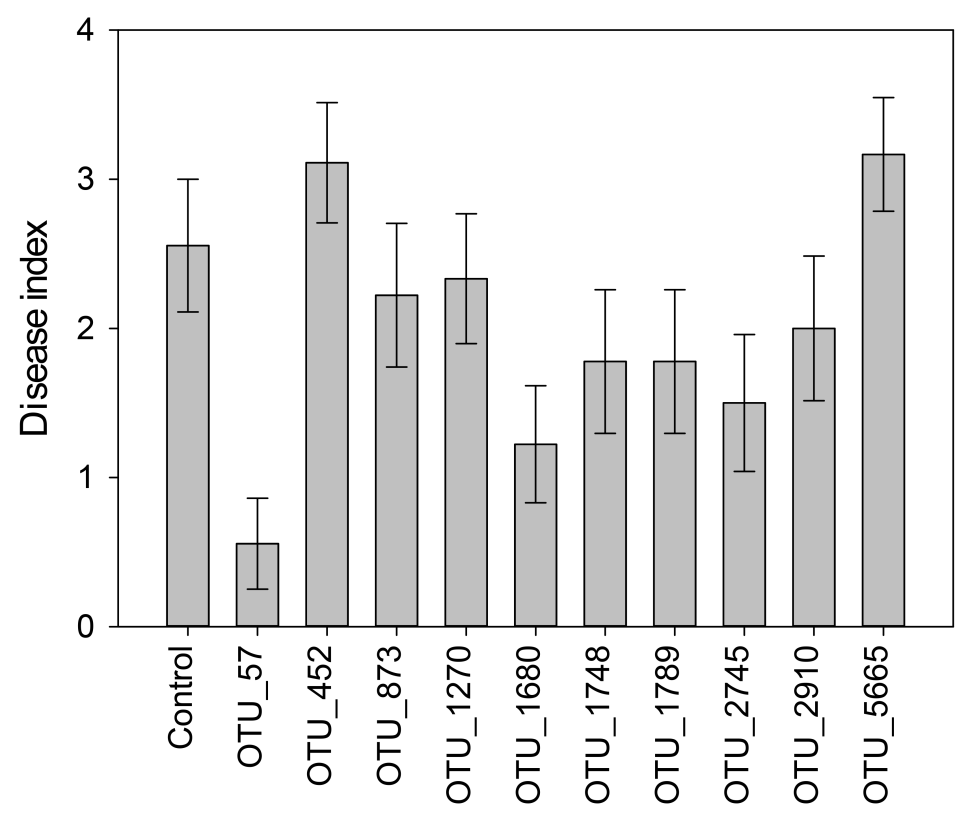


**Fig. S6 Effects of ten non-discriminating OTUs between healthy and diseased plants (corresponding to Fig. 4F) at VS2 on of disease index of bacterial wilt (*n* = 18).**

**
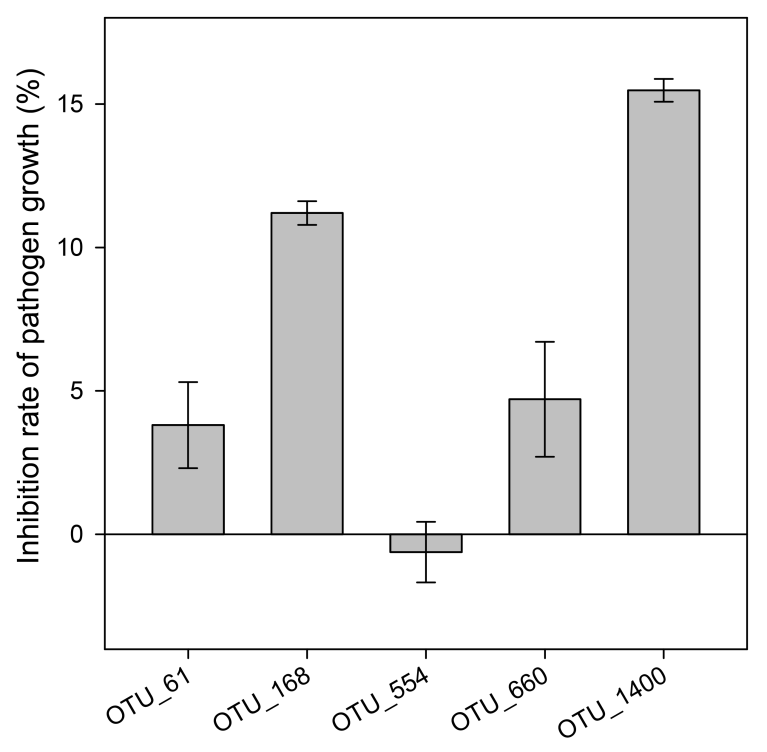
**

**Fig. S7 Growth rate inhibition of *R. solanacearum* by the five healthy-plant-enriched OTUs (*n* = 3).**

**
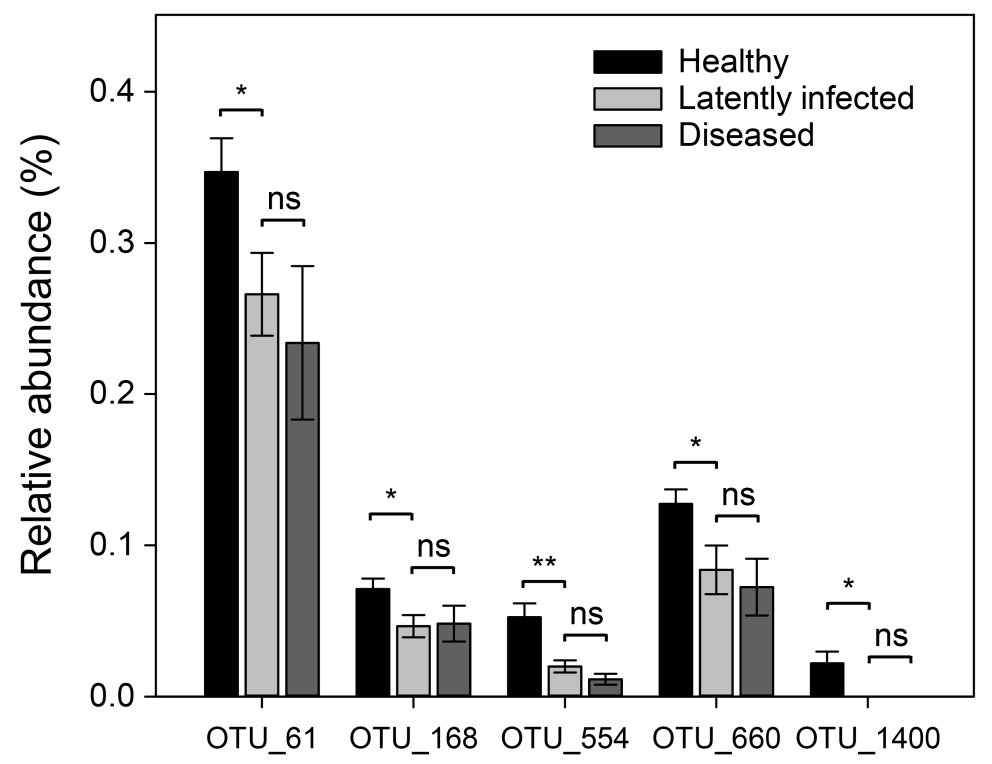
**

**Fig. S8 Relative abundance of the five healthy-plant-enriched OTUs in the rhizosphere of healthy, latently infected, and diseased plants at vegetative stage 2.** *, *p* < 0.05; **, *p* < 0.01.
